# Analyzing Dropout in Alcohol Recovery Programs: A Machine Learning Approach

**DOI:** 10.3390/jcm13164825

**Published:** 2024-08-15

**Authors:** Adele Collin, Adrián Ayuso-Muñoz, Paloma Tejera-Nevado, Lucía Prieto-Santamaría, Antonio Verdejo-García, Carmen Díaz-Batanero, Fermín Fernández-Calderón, Natalia Albein-Urios, Óscar M. Lozano, Alejandro Rodríguez-González

**Affiliations:** 1 CentraleSupélec, Université Paris-Saclay, 91190 Gif-sur-Yvette, France; 2 Centro de Tecnología Biomédica, Universidad Politécnica de Madrid, 28223 Pozuelo de Alarcón, Spain; 3 Escuela Técnica Superior de Ingenieros Informáticos, Universidad Politécnica de Madrid, 28660 Boadilla del Monte, Spain; 4 Turner Institute for Brain and Mental Health, Monash University, Melbourne, VIC 3800, Australia; 5 Clinical and Experimental Psychology Department, University of Huelva, 21071 Huelva, Spain; 6 Research Center for Natural Resources, Health and the Environment, University of Huelva, 21071 Huelva, Spain; 7 Discipline of Psychology, Federation University, Berwick, VIC 3806, Australia

**Keywords:** alcohol use disorder, treatment, outpatients, machine learning, outcomes, dropout, real-world data

## Abstract

**Background**: Retention in treatment is crucial for the success of interventions targeting alcohol use disorder (AUD), which affects over 100 million people globally. Most previous studies have used classical statistical techniques to predict treatment dropout, and their results remain inconclusive. This study aimed to use novel machine learning tools to identify models that predict dropout with greater precision, enabling the development of better retention strategies for those at higher risk. **Methods**: A retrospective observational study of 39,030 (17.3% female) participants enrolled in outpatient-based treatment for alcohol use disorder in a state-wide public treatment network has been used. Participants were recruited between 1 January 2015 and 31 December 2019. We applied different machine learning algorithms to create models that allow one to predict the premature cessation of treatment (dropout). With the objective of increasing the explainability of those models with the best precision, considered as black-box models, explainability technique analyses were also applied. **Results**: Considering as the best models those obtained with one of the so-called black-box models (support vector classifier (SVC)), the results from the best model, from the explainability perspective, showed that the variables that showed greater explanatory capacity for treatment dropout are previous drug use as well as psychiatric comorbidity. Among these variables, those of having undergone previous opioid substitution treatment and receiving coordinated psychiatric care in mental health services showed the greatest capacity for predicting dropout. **Conclusions**: By using novel machine learning techniques on a large representative sample of patients enrolled in alcohol use disorder treatment, we have identified several machine learning models that help in predicting a higher risk of treatment dropout. Previous treatment for other substance use disorders (SUDs) and concurrent psychiatric comorbidity were the best predictors of dropout, and patients showing these characteristics may need more intensive or complementary interventions to benefit from treatment.

## 1. Introduction

Alcohol consumption is a leading cause of more than 200 diseases, injuries, and health conditions. Excessive and hazardous alcohol use poses a significant risk for the development of mental and behavioral disorders, including alcohol use disorder (AUD), as well as major noncommunicable diseases. To reduce harmful alcohol use in line with Sustainable Development Goals (SDGs) 2030 targets and the WHO Global Monitoring Framework, new research on preventive, harm reduction, and treatment pathways which engages key stakeholders (e.g., government treatment networks) is crucial [1].

In regard to treatment seeking for drug abuse, alcohol is either the primary or secondary drug for almost half a million people in the USA [2]. In the case of Spain, which is one of the European countries that systematically collects information on addiction treatment admissions, over one in three admissions are due to alcohol [3]. Although there is a range of evidence-based treatments for AUD [4,5,6], they have pervasive limitations, particularly their low retention and engagement rates [7]. Specifically, both nation-wide and international studies show that more than half of the patients undergoing treatment had previously received treatment for their addiction [2,3].

From an epidemiological perspective, there are several correlates (medical, psychological, social) associated with AUD that hamper engagement and ultimately recovery [8]. These variables have also been integrated into theoretical models to explain their relationships during the therapeutic process [9,10]. These models indicate that time in treatment, as well as premature dropouts, have a negative impact on the patient’s therapeutic prognosis. In fact, more than half of readmissions to treatment occur with patients who have dropped out of treatment without achieving the therapeutic objectives [11,12]. For this reason, reducing dropouts in treatment is one of the clinical and managerial targets to achieving a more efficient and effective treatment.

As some authors point out [13,14], machine learning (ML) techniques can provide strong benefits in the substance abuse field relative to traditional techniques, as they can uncover hidden relationships in the data, which can help to improve clinical decisions. They have the potential to identify if a given patient could drop out from the therapeutic process, and consequently, to trigger additional support for those users with a higher likelihood of dropout. The creation of ML models not only will allow the potential use of such models but also assist in seeing what are the most relevant features that are influencing a concrete output. This can help in having a better understanding of what the factors (psychological, clinical, societal, etc.) driving the decision to drop out are, and can help professionals in providing better patient care.

In recent years, various studies have applied ML techniques to predict dropouts and relapses in the context of addiction treatment. So far, studies with a sampling ranging from 275 to 1383 patients [15,16,17,18,19,20] have used different techniques of ML to predict AUD treatment outcomes. According to the authors of [13], the ML method demonstrated its superiority in predicting outcomes compared to other techniques. However, they also suggest important limitations associated with the ecological validity of these techniques.

In this context, for those models typically considered black-box models (not providing interpretability), a set of techniques to try to provide model explainability will be applied. In this regard, the main aims of this study are as follows: (i) to study different types of machine learning algorithms over a dataset of patients that have discontinued (or not) the therapeutic process, to investigate their usefulness in identifying patients with a higher risk of dropping out; and (ii) to pinpoint the most relevant variables predicting treatment dropout in AUD patients.

## 2. Material and Methods

### 2.1. Dataset Description

The original dataset consisted of 39,030 patients with AUD. All patients started treatment at any of the 121 public and subsidized addiction treatment centers in Andalusia (a region of Spain with more than 8,000,000 inhabitants) from 1 January 2015 to 15 September 2021. From this dataset, patients with no treatment completion data were excluded (7943 patients), as they could not be used in the training phase because we do not know the output of the therapeutic process. The data for the analysis contained a total of 31,087 patients.

The data utilized in this study are derived from the Electronic Health Records (EHRs) of the Information System of the Andalusian Plan on Drugs (SiPASDA), which maintains a centralized dataset for all addiction centers. The EHR stores various information following the standards outlined by the European Monitoring Centre for Drugs and Drug Addiction [21]. This includes sociodemographic variables, drug use history, previous treatments, and infectious diseases. The SiPASDA data have been used in previous studies, demonstrating the reliability of the records [22], as well as their utility for understanding the evolution of patients and their therapeutic outcomes during treatment [9,11,23].

The storage and encoding of these data adhere to the General Health Law dated 25 April 1986 (Spain), as well as Law 41/2002 passed on November 14, which pertains to patient autonomy, rights, and obligations regarding clinical information and documentation. Furthermore, it complies with the Organic Law 3/2018 enacted on 5 December 2018, which focuses on safeguarding personal data and ensuring digital rights in accordance with European regulations. 

The Andalusian Ministry of Health’s Research Ethics Committee confirmed that the information was handled in accordance with its ethical standards.

### 2.2. Methodological Approach

The methodology employed in the analysis and model creation is based on the Cross-Industry Process for Data Mining (CRISP-DM) [24], a comprehensive framework for developing machine learning models. This methodology encompasses phases from business understanding to deployment, ensuring a systematic approach. The primary business objective is to reduce the dropout rate from addiction treatment programs by predicting patients at a high risk of early departure. The initial data comprised 215 variables and was reduced to 129 through filtering out irrelevant or highly null variables. A further dimensionality reduction involved clustering mental disorders and applying a gain ratio for variable selection, resulting in a final dataset of 19 predictors. Multiple machine learning algorithms, including logistic regression, support vector classifiers, decision trees, random forests, bagging, boosting, and neural networks, were evaluated using 10-fold cross-validation. Class imbalance was addressed through oversampling techniques like SMOTE and Tomek links. Hyperparameter tuning was performed via randomized search, optimizing for precision to minimize false positives. Model explainability was enhanced using SHAP values to interpret the influence of input features. The entire process was executed using R and Python, with experiments conducted on a high-performance computing setup, and all results were made publicly available to ensure reproducibility. The following subsections explain the different steps that belong to this methodology in more detail. Finally, in the available online (https://medal.ctb.upm.es/internal/gitlab/compara/mldropoutalcohol accessed on 12 August 2024) repository, all the relevant details of each CRISP-DM phase are described.

*Business understanding*: The main business objective is to reduce the dropout rate of patients from addiction treatment programs by identifying individuals at a higher risk of leaving the treatment early. This can help in customizing interventions, improving patient engagement, and ultimately enhancing treatment outcomes. Understanding the factors that contribute to treatment discontinuation can also inform program improvements and policy adjustments. Hence, the aim of the associated data mining is to develop a predictive model that can estimate the likelihood of a patient discontinuing their addiction treatment.

#### 2.2.1. Data Understanding and Data Preparation: Variables Description and Dataset Creation

The initial dataset contained a total of 215 variables, this being a high-dimensional dataset. In order to reduce the complexity of the problem and maximize the inclusion of variables with a higher potential for predictive purposes, a detailed analysis of the available variables was performed to select the most relevant ones. First, we only kept those variables with values in at least 60% of the rows (discarding those with >40% of null values). Additionally, categorical data features were retained if the number of possible values for those features was less than 20 values, to maintain manageable and meaningful data representations. Other potential variables without predictive value (such as Identifiers (IDs)) were also removed. After this process, the performed reduction resulted in a new dataset with a total of 129 variables and the aforementioned 31,087 patients.

##### Dimensionality Reduction: Mental Disorder Clustering

A second dimensionality reduction process was also performed, considering as input the 129 variables and the 31,087 patients derived from the previous process. In this case, mental disorders were grouped into more general categories that integrated disorders with close descriptive similarities (categories of disorders from the ICD-10 classification). This second process led to creating a second dataset with a total of 50 variables and 31,087 patients.

##### Dimensionality Reduction: Variable Filtering

A second step to reduce the dimensionality of the dataset and to try to obtain better predictors was carried out by performing variable filtering. In this case, a subset of the variables was selected using a univariate filtering approach named gain ratio [25], a metric derived from their mutual information. This method is a specific instance of a broader concept known as relative entropy. Mutual information can be seen as the reduction in uncertainty about a given variable after observing another variable. The information gain ratio technique normalizes mutual information by considering the entropy value of each variable. This is necessary, since mutual information favors variables with many different values.

The top 20 predictor variables ranked by the gain ratio between the variables and the output became part of the selected feature subset. It is important to note that univariate approaches do not measure correlation. Therefore, to eliminate highly correlated variables, the correlation matrix between the features was analyzed, and variables with a correlation coefficient greater than 0.75 were excluded. By using this method, a total of 19 variables were selected, irrespective of the original dataset. The concrete list of variables selected by each subdataset (cluster/no cluster) is available online (https://medal.ctb.upm.es/internal/gitlab/compara/mldropoutalcohol/blob/master/results/filtering_discretization.txt accessed on 12 August 2024). The selected variables can be classified into the following categories:

- Consequences of alcohol consumption on the patient’s life, namely, road accidents while intoxicate, administrative sanctions, work absenteeism, loss of employment, conflicts with partners, and other problems that are due to alcohol use (e.g., legal problems, risk of social exclusion);
- Addictions other than alcohol use disorders, such as abuse or dependence on hypno-sedatives, stimulants other than cocaine, pathological gambling, and whether the patient has already received opioid substitution treatment;
- Comorbidity with mental disorders other than addictions;
- Personal details of the patient’s living arrangements, such as where they have lived or whether they live with family members with addictions;
- Physical health indicators such as transaminase levels, which can be a symptom of liver disease;
- Previous treatment for addictions and mental health.

**Figure 1:**
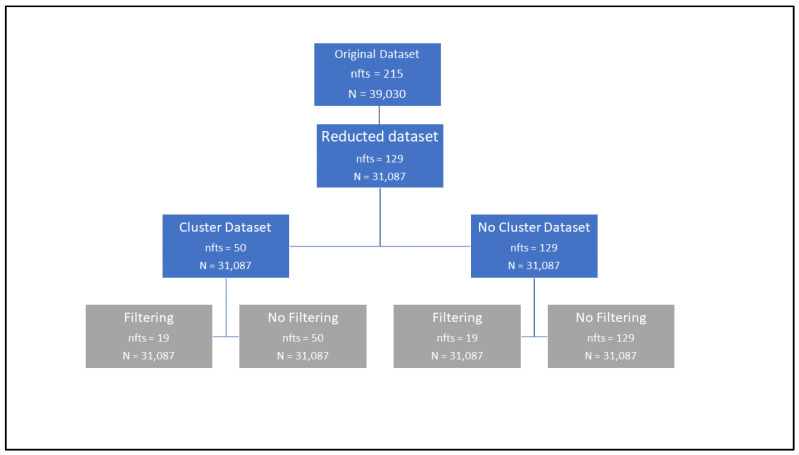
Datasets generated depending on the application of clustering and filtering.

Finally, to have a better understanding of the results derived from the “data understanding/data preparation” process, including the reduction of the dimensionality of the dataset by applying the typical dataset preparation (Section 2.2.1), the clustering process (first subsection of Section 2.2.1), and the variable filtering (second subsection of Section 2.2.1), Figure 1 is shown.

Figure 1 shows the different datasets that were generated. In the root, we have the original dataset (number of features (nfts) = 215; population (N) = 39,030). The second-level dataset corresponds to the first filtering (number of features (nfts) = 129; population (N) = 31,087). That is, after removing irrelevant variables (those with a high number of null values/categories or without semantic predictive potential), we have our “Reducted dataset”. The third-level datasets are the cluster dataset (which is the one where the mental disorders were clustered (leading to 50 features)) and the no cluster dataset (where the original specific mental disorders were maintained (maintaining 129 features)). In the leaf nodes (in gray), we have the final 4 datasets that were used for the creation of the machine learning models. Filtering and no filtering are explained in the next section.

### 2.3. Outcome

The dependent variable was discontinuation of treatment. A patient was considered to have abandoned treatment when, according to clinical judgement, they had not achieved their therapeutic goals and had not visited the treatment center for more than six months.

In the final dataset of 31,087 patients, 78% of them had eventually dropped the treatment, while 22% of them successfully completed it. This imbalance between the two classes of the outcome variable has been addressed as described in the following section.

### 2.4. Model Selection and Training

We conducted an in-depth analysis, comparing different algorithms to ensure that we identify the best option for our aim of predicting discontinuation of the therapeutic process [26]. In this case, we have considered 7 algorithms for evaluation.

- Simple models: The first group consisted of three “simple” models, namely logistic regression, support vector classifier (SVC), and classification tree;
- Meta-classifiers: Additionally, three meta-classifiers, namely random forest, bagging, and boosting, using decision trees as an estimator, were utilized;
- Neural networks: Lastly, a neural network model in the form of a multi-layer perceptron (MLP) was also employed to complete the set of selected models.

The training and evaluation process has been carried out using a 10 K-fold cross-validation strategy, where each fold is used once as the testing subset, and the remaining folds are used for training the model. This approach involves dividing the dataset into 10 equally sized folds or subsets, ensuring that each fold serves as the testing subset exactly once during the cross-validation process. To address the class imbalance during the training of the models, we considered the use of oversampling techniques. Although the percentage of elements in the minority class (22%), considering the sample size (31,087), was enough to consider the application of an undersampling over the majority class, we have considered oversampling to addresses class imbalance by augmenting the minority class, thereby preserving valuable information and enhancing the model’s performance without the loss of critical data inherent in undersampling strategies. Concretely, the Synthetic Minority Over-Sampling Technique (SMOTE) [27] and the Tomek links [28] methods were employed to balance the classes by creating synthetic instances over the minority class until reaching a balance between both classes. It is noteworthy that these sampling techniques were performed within each fold of the training process to mitigate the risk of overfitting and ensure a more robust model evaluation. By balancing the classes and mitigating bias during the training phase, the model can better capture the characteristics of both the majority and minority classes. This leads to more accurate predictions, improves overall performance, and reduces the potential for biases.

### 2.5. Model Creation

The hyperparameter tuning was conducted using randomized search, with the objective of optimizing the precision score. Precision is calculated following this formula:

$$Precision=\frac{True\;positive\;values}{True\;positive\;values+False\;positive\;values}$$

This approach involves randomly sampling combinations of hyperparameters within specified ranges, to find the set of hyperparameters that maximizes the performance metric. Precision was chosen as a metric, since it limits the number of false positives (negative samples incorrectly classified as positive). This means that if a patient is classified as a potential future dropout by our models, they have a very high probability of being one and as such require special attention.

To evaluate the models, we will use the precision score averaged over the 10 folds of the training process and then compute the 95% confidence interval with the standard deviation of each series. Two models with overlapping confidence intervals cannot be distinguished in terms of performance and will therefore be considered equivalent. This helps to ensure that the difference in performance is not due to chance and reflects the intrinsic qualities of each model.

### 2.6. Explainability Methodology

Since meta-classifiers MLP and SVC are black-box models, the SHAP (SHapley Additive exPlanations) approach is applied to give an insight into the inputs’ influence over the decision [29]. SHAP is a game-theoretic method that provides explanations for the output of any machine learning model. By leveraging classic Shapley values from game theory and their related extensions, SHAP connects the optimal credit allocation with local explanations. This approach allows for a comprehensive understanding of how inputs contribute to the model’s output, revealing their specific influence on the final predictions. 

Two types of graphs are generated to analyze the results of the SHAP computation: (1) The first is a bar graph showing the absolute Shapley value associated with each input. It displays the most important ones, no matter whether their influence on the prediction is positive or negative. And (2), the second is a scatter graph presenting the distribution of the values taken by each input on the Shapley values scale, each dot representing one patient. It explains how each feature influences the prediction.

### 2.7. Experimental Setup

We used different procedures for different aspects of data processing and model creation. Concretely, the feature engineering was performed using R 4.1.0, while the machine learning was conducted using Python 3.8.10. The experiments were conducted using the CUDA Toolkit 11.3 on an Ubuntu Server LTS 20.04.4 with a GPU (NVIDIA GeForce RTX 3090 24 GB), Intel i9-12900K, and 32 GB RAM. To ensure reproducibility, all the materials and results have been published in an online repository (https://medal.ctb.upm.es/internal/gitlab/compara/mldropoutalcohol accessed on 12 August 2024).

## 3. Results

### 3.1. Model Selection

The training of all seven models was carried out on the four datasets, to determine which dataset and algorithm produced the best output score. The objective was to identify the model with the highest precision score, and, in the case of similar results, use F1 and recall scores as tie-breakers. The results of the best performing model on all four datasets are summed up in Table 1. A detailed spreadsheet with the results of all the tested algorithms across the four datasets is available online (https://medal.ctb.upm.es/internal/gitlab/compara/mldropoutalcohol/blob/master/results/models_results.xlsx accessed on 12 August 2024).

Two datasets stood out for their high precision score: the one with clustering but no filtering, with a score of 0.825 ± 0.006, and the one with no clustering but filtering, with a score of 0.824 ± 0.002. As the difference in precision is not significant, we compared the *F*1 and recall scores to choose the best one (filtering but no clustering). The recall and *F*1 (considering current recall and previous precision formulas) formulas are defined below:

$$Recall=\frac{True\;positive\;values}{True\;positive\;values+False\;negative\;values}$$

$$F1-score=\frac{2*Precision*Recall}{Precision+Recall}$$

**Figure 2:**
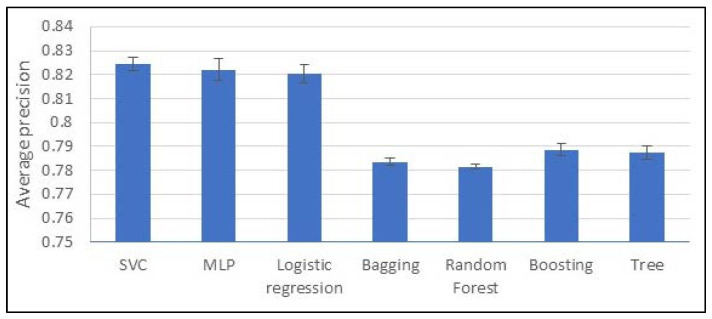
Average precision of models over 10-fold training and non-clustered dataset.

Regarding the model selection on the filtered and non-clustered dataset, the three best-performing models have an overlapping confidence interval (see Figure 2). Consequently, their difference in performances is not statistically significant. However, their precision scores are significantly better than all the other models. As a matter of fact, we can conclude that SVC, MLP, and logistic regression are the best choices out of the seven tested. They have a precision ranging from 0.824 ± 0.002 to 0.8204 ± 0.003, an F1 score between 0.671 ± 0.006 and 0.64 ± 0.02, and a recall score from 0.567 ± 0.007 to 0.533 ± 0.008.

### 3.2. Performance of the Model in a Real Scenario

We also conducted one extra testing, to assess the models’ performance in a real-world case scenario. To do so, we separated a set of data (10% of the entire dataset) at the first stage of the learning process, which was reserved just for computing the final evaluation metrics. No sampling or parameter tuning was carried out from this test set. We performed this external evaluation in the four pipelines previously described: (i) without filtering variables and without clustering, (ii) without filtering the variables but clustering them, (iii) filtering the variables but without clustering them, and (iv) filtering and clustering the variables. The results are displayed in Table 2. We implemented this process with the SVC method, given that it was the one generally working better in the previous testing.

### 3.3. Explainability Results

**Figure 3:**
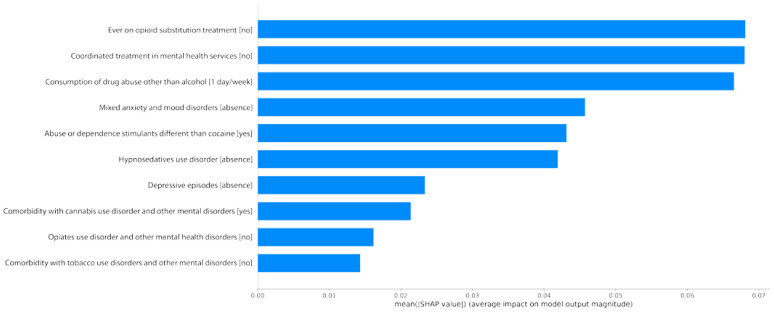
Ten most influential variables on the SVC model with the filtered/non-cluster dataset.

For the sake of simplicity, the explainability study was conducted on the best performing combination of model/dataset: the SVC algorithm with the filtered/non-cluster dataset. Figure 3 shows a bar graph with the 10 most influential features.

The average importance of a variable is about 0.05, implying that the first three features have a greater-than-average performance. The next three are in the expected range, and the last four are above average. The remaining nine features are thus not shown, since we want to focus on the most influential ones.

The SHAP value plot illustrates key variables influencing the likelihood of a patient abandoning alcohol abuse treatment. Notably, the absence of previous opioid substitution treatment and coordinated mental health services are significant predictors. These factors suggest that patients who have not experienced structured, integrated care are more prone to treatment dropout. Additionally, the regular consumption of other drugs and the presence of stimulant abuse highlight the complexities of poly-substance use, which can undermine the focus and success of alcohol-specific treatment.

Mental health variables also play a critical role. The absence of mixed anxiety and mood disorders, depressive episodes, and hypnotics/sedative use disorders might indicate a lack of comprehensive mental health support, making it easier for patients to disengage from treatment. Conversely, comorbid conditions like cannabis use disorder add complexity, increasing the likelihood of treatment abandonment. Overall, these variables underscore the importance of integrated and multifaceted treatment approaches, to enhance adherence and reduce dropout rates in alcohol abuse programs.

**Figure 4:**
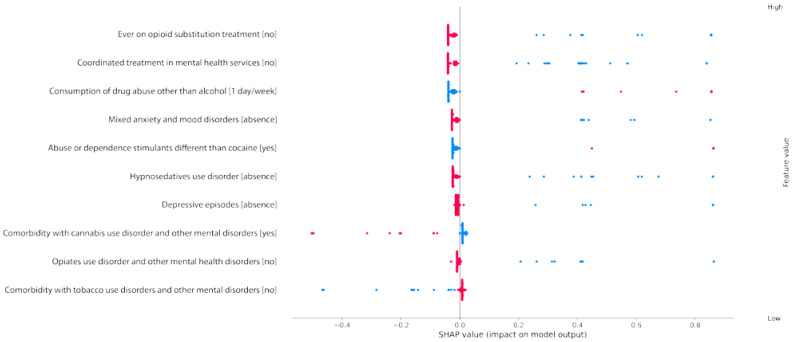
Graph with the distribution of input values as a function of the associated Shapley value (red implies a high value for the feature; blue implies a low value for the feature).

To further analyze their influence over the prediction, Figure 4 presents a scatter graph showing the distribution of input values as a function of the associated Shapley value. It mainly leads to interpreting each input as being positively or negatively correlated with the output.

From these results, we can infer how each variable influenced the prediction and to what extent. Figure 4 represents in red the instances that have a specific value [value indicated between brackets] next to the variable. Blue dots represent the instances that take the other possible value for each variable. The fact that the patient had ever received opioid substitution treatment (blue dots in the first row in Figure 4) is highly correlated with the output, which means that a patient who has ever received such treatment is deemed by our model to have a high probability of dropping out. On the other hand, having not received this treatment before has almost no influence in the model towards classifying a patient as to not dropping out. In other words, on the one hand, the points associated with a patient that has never had a substitution treatment (red points) are very close to the origin of the plot. They range from −0.05 to 0, which means that they have a marginal negative influence over the prediction of someone as a dropout. On the other hand, the points associated with patients that have had an opioid-based substitute treatment (blue points) have a scarce distribution ranging from 0.3 to 0.8. Indeed, having received such treatment in the past has a lot of influence over the classification of the patient as a dropout.

A similar interpretation can be drawn for most of the other inputs considered. On the one hand, a patient who is simultaneously undergoing a treatment for mental disorders has a very high chance of being a dropout. On the other hand, not following such a treatment does not have an influence in the model to classify the patient as to not dropping out. Similarly, a patient who consumes another drug once a week has a very high chance of being classified as a dropout, but not doing so is not correlated with dropout probability. In a similar way, patients who have anxiety disorders, depressive episodes, or disorders due to the use of hypno-sedatives are considered by the model as at a very high risk of dropping out. However, not presenting such behaviors has only an unimportant influence on lowering the estimated dropout rate. Out of the ten most influential inputs, two of them actually help to characterize patients who will not drop out. These are the eighth and tenth criteria: disorders due to cannabis and tobacco consumption. This time, the distribution is scarce on the negative Shapley value half-plane and compact close to zero on the positive Shapley value half-plane. Consequently, the model interprets having cannabis or tobacco-related disorders as a sign that the patient is at a significantly lower risk of dropping out. However, the model does not interpret not presenting these addictions as very significant.

## 4. Discussion

This study applied machine learning techniques to identify predictors of treatment discontinuation in an unprecedentedly large sample of 30,000 participants monitored via a state-wide health information system. This unique sample allowed us to directly compare the utility of different cutting-edge machine learning approaches, as well as identifying variables related to alcohol consumption, other substance use, and psychiatric comorbidities as key predictors of treatment discontinuation.

Firstly, there are different ML algorithms or techniques that can help to create models to discern whether or not a patient is going to abandon their therapeutic process. The use of a large dataset allowed us to effectively compare several different algorithms, and to identify those with a better precision in outcome prediction. This through-comparison and selection process facilitates translation potential, as the identified algorithm is likely to predict clinical outcomes with a minimal risk of false positives. On the other hand, the dataset and analytic approach also enabled us to effectively combine black-box algorithms with explainability approaches. This combination marries the computational advantages of SVC with the explanatory power of SHAP and yields both a high precision and conceptual value by qualifying the most meaningful predictors and their directionality. This combo approach may enable new applications of black-box algorithms. As seen in the present study, highly complex algorithms such as the multi-layer perceptron (MLP) or support vector classifier (SVC) generally perform well in identifying patients who will potentially drop out of treatment. The accuracy measures, which allow us to measure the proportion of true positive predictions, are above 80%.

Moreover, explainability is an important factor in these models. Given that the models that have obtained the best efficiency are black-box models, this manuscript presents an analysis of their main characteristics through the SHAP approach, which allows a clearer understanding of the main variables that are influencing the model. This methodological approach is novel with respect to existing studies that apply artificial intelligence techniques to predict a therapeutic outcome in substance use disorder patients [30]. In addition, its extrapolation to other treatments for patients with mental health problems in general, where high rates of treatment abandonment and non-compliance with medical prescriptions are observed, should be assessed [31].

Specific predictors included a prior history of opioid substitution treatment (OST) and comorbidities with other substance use and mental disorders. Together, these findings suggest that participants with AUD, plus other manifestations of mental ill-health, are the ones at a higher risk of discontinuation of treatment, and in need of adjunctive or more intensive interventions. The impact of OST was unexpected and probably reflects the influence of a history of opioid use disorder, which is also consistent with the predicting relevance of other drug use, as other studies have shown [32]. The impact of the concurrent use of mental health services suggests that a broader and more intense psychopathology profile is linked to risk of discontinuation of addiction treatment. In this regard, the findings of machine learning methods align with the classic clinical literature regarding the influence of comorbidities and clinical “complexity” [33].

With respect to this, we believe that studying these patients using dimensional models, such as the Hierarchical Taxonomy of Psychopathology [34] of the Research Domain Criteria [35], could better capture these psychopathological characteristics and be useful for predicting treatment retention. This also aligns with current policy discussions, such as which is the best approach for treating addictive disorders (i.e., parallel or integrated pathways with mental health services). In this vein, several studies have shown that people with alcohol and other substance use disorders and mental health comorbidities spend less time in treatment and have a greater risk of discontinuation. However, those patients with comorbidities that undergo customized integrated treatments can achieve a similar therapeutic success to patients without comorbidities. Thus, the present study, which was conducted on a large sample using real-world data, seems to reflect that the current mainstream treatment approach may not be catering to the needs of patients with comorbidities,

Despite our large sample and innovative approach, we should also acknowledge some limitations. Firstly, the sample was mainly composed of male participants. This probably reflects the uneven distribution of gender in the target population, as reflected in several epidemiology reports [36]. However, it also indicates that new efforts are needed to better understand predictors of treatment outcomes, specifically among women. Regarding the limitations of the analyses, it should be noted that due to the existing imbalance between classes, the application of balancing techniques always implies a decrease in instances of a class (undersampling) or the synthetic generation of the minority class (oversampling). Although, as mentioned in this article, the use of these techniques was used to mitigate the risk of overfitting and ensure a more robust model evaluation, it is true that having a more balanced dataset from the original data will always produce better results.

In terms of results, the approach used has allowed us to discern, using variable filtering techniques and clustering approaches, which is the best potential dataset and the best technique that offers the most robust results for the detection of dropout. Although the results are promising and it is considered that a series of models with high precision values have been obtained, future research where access to other types of variables that could improve the characterization of the patient, in order to define their potential dropout likelihood, would be very useful.

Regarding the limits of the SHAP algorithm, one of the main assumptions made is that the variables are independent. Since the filtering applied to the dataset requires the highly correlated variables to be excluded, the remaining ones have a low correlation rate. However, this does not mean that they are independent stricto sensu. Furthermore, one should bear in mind that SHAP is not a measure of the importance of the variables in a real-life phenomenon but rather an insight into the functioning of a machine learning algorithm. Such models find patterns in data, and the SHAP analysis highlights them. However, the trends observed do not demonstrate in any way a causal link between a variable and a phenomenon.

A final comment on this section needs to be made with respect to the separation of the dataset in the training and test sets. We acknowledge the importance of strictly separating the training and test data before performing steps such as feature selection, normalization, imputation, hyperparameter tuning, and model selection. We understand that this is a critical aspect of preventing data leakage and ensuring the generalizability of the model. However, in our case, we did not strictly adhere to the pre-split procedure; we have taken several measures, including the additional experiments shown in Section 3.2, to mitigate potential biases and ensure the robustness of our model. Concretely, we would like to emphasize the importance of employing cross-validation techniques to evaluate the model’s performance. This method involves dividing the data into multiple folds, ensuring that the training and test data are separated within each fold. Although this does not replace a strict pre-split, it does provide a robust mechanism to assess the model’s generalizability across different subsets of the data. On the other hand, the feature selection process was guided by domain knowledge and statistical analyses independent of the model training process. While this was carried out before the splitting, the choice of features was not influenced by the test data but was based on their inherent characteristics, thus reducing the risk of overfitting. Finally, these steps were performed using parameters derived from the entire dataset. While ideally carried out post-split, we believe the impact on the model’s performance is minimal due to the cross-validation process, which ensures that the test data in each fold remain unseen during the model’s fitting phase. We recognize the ideal methodological approach would have involved a pre-split of the data. However, given the constraints and the data availability at the time, we prioritized a methodologically sound approach using cross-validation, which is widely accepted as a rigorous evaluation method, and we performed the additional experiments provided in Section 3.2 to increase the validity of the results.

## 5. Conclusions

In conclusion, our machine learning approach to predicting treatment dropout for alcohol use disorder (AUD) demonstrates significant potential for a broader application across various health conditions. This methodology can be effectively extended to other substance use disorders, chronic diseases such as diabetes and hypertension, mental health disorders, rehabilitation programs, and preventive health initiatives. By accurately identifying patients at a higher risk of discontinuing treatment, we can develop and implement targeted retention strategies, thus improving treatment adherence and outcomes across diverse medical and therapeutic contexts. This highlights the versatility and practical utility of our approach in enhancing patient care and treatment success.

## Figures and Tables

**Table 1:** Precision of the dataset generated.

		No Clustering	Clustering
No filtering	Precision	0.811	0.825
	F1	0.242	0.604
	Recall	0.142	0.477
	Model	Support vector classification	Support vector classification
Filtering	Precision	0.824	0.799
	F1	0.648	0.639
	Recall	0.534	0.557
	Model	Support vector classification	Multi-layer perceptron

**Table 2:** Performance of the models in a real scenario.

		No Clustering	Clustering
No filtering	Precision	0.777	0.777
	F1	0.874	0.874
	Recall	0.998	1
	Model	Support vector classification	Support vector classification
Filtering	Precision	0.811	0.789
	F1	0.237	0.691
	Recall	0.139	0.615
	Model	Support vector classification	Support vector classification

## Data Availability

All additional data is available at: https://medal.ctb.upm.es/internal/gitlab/compara/mldropoutalcohol (accessed on 13 August 2024).
